# Parameter optimization for automated behavior assessment: plug-and-play or trial-and-error?

**DOI:** 10.3389/fnbeh.2014.00028

**Published:** 2014-02-04

**Authors:** Laura Luyten, Natalie Schroyens, Dirk Hermans, Tom Beckers

**Affiliations:** ^1^Psychology of Learning and Experimental Psychopathology, Faculty of Psychology and Educational Sciences, KU LeuvenLeuven, Belgium; ^2^Experimental Neurosurgery and Neuroanatomy, Department of Neurosciences, KU LeuvenLeuven, Belgium

**Keywords:** freezing, fear conditioning, rats, automated measurements, parameter optimization, calibration, manual scoring, VideoFreeze

## Abstract

Behavioral neuroscience is relying more and more on automated behavior assessment, which is often more time-efficient and objective than manual scoring by a human observer. However, parameter adjustment and calibration are a trial-and-error process that requires careful fine-tuning in order to obtain reliable software scores in each context configuration. In this paper, we will pinpoint some caveats regarding the choice of parameters, and give an overview of our own and other researchers' experience with widely used behavioral assessment software. We conclude that, although each researcher should weigh the pros and cons of relying on software vs. manual scoring, we should be aware of possible divergence between both scores, which might be especially relevant when dealing with subtle behavioral effects, like for example in generalization or genetic research.

## Introduction

Over the years, fear conditioning research in rodents has moved from purely “manual” scoring of freezing behavior by human observers to mainly automated measurements. Since a few years, conditioning researchers have (re)focused on generalization, including generalization of context conditioning, because of its relevance to the study of learning and memory, and to the development and maintenance of anxiety disorders (Wang et al., 2009; Wiltgen et al., 2010; Hermans et al., 2013). Contextual generalization/discrimination research necessitates that freezing in several contexts is compared directly (e.g., Wang et al., 2009; Wiltgen et al., 2010; Yu et al., 2010). Counterbalancing of contexts is not always possible, e.g., in case of a generalization gradient with some contexts having a grid and others a plastic floor (Luyten et al., 2013; Poulos et al., 2013). Furthermore, the differences in freezing between the originally trained and a similar context are often modest, and not as clear-cut as between the original and a dissimilar context (unpublished data), resulting in limited effect sizes. Concurrently, there is an increasing interest in transgenic animals, to investigate the role of certain genes in discrimination and generalization (e.g., Yu et al., 2010; Tayler et al., 2011; Cushman et al., 2012). Genetic modifications may, however, result in phenotypes with only small behavioral deficits.

Taken together, this growing domain is confronted with the challenge to distinguish subtle behavioral effects in different contexts. Our data show that automated behavioral measurements may not always be appropriate for these purposes, or that, at least, they should be implemented and interpreted with great care.

To our knowledge, there is only one paper that has systematically validated settings for a widely used system to detect freezing in rodents (i.e., Med Associates, Inc. Video Fear Conditioning System, with VideoFreeze^®^ software). The optimized software parameters for freezing mice [motion index threshold 18 and minimum freeze duration 30 frames (1 s)], were published in this journal (Anagnostaras et al., 2010). One of the challenges of parameter optimization is finding a balance between the detection of “non-freezing” (e.g., tail) movements, while at the same time ignoring respiratory and cardiac motion during freezing. VideoFreeze is also frequently used to assess rat freezing behavior and others have reported their validated settings for rats (motion threshold 50) (Zelikowsky et al., 2012a). It makes sense to use a higher activity threshold for rats, as they are bigger animals and respiratory movements—which do not preclude freezing—will result in more pixels changing than for mice. Those are also the settings that we used in our studies.

## Materials and methods

Experiments were conducted on 48 male Wistar rats (±275 g), in three replications of 16 rats each (eight rats per group). All sessions were meticulously scheduled using free ExpTimer software (Luyten and Van Cappellen, 2013). All experiments were approved by the KU Leuven animal ethics committee, in accordance with the Belgian Royal Decree of 29/05/2013 and European Directive 2010/63/EU.

**Figure 1:**
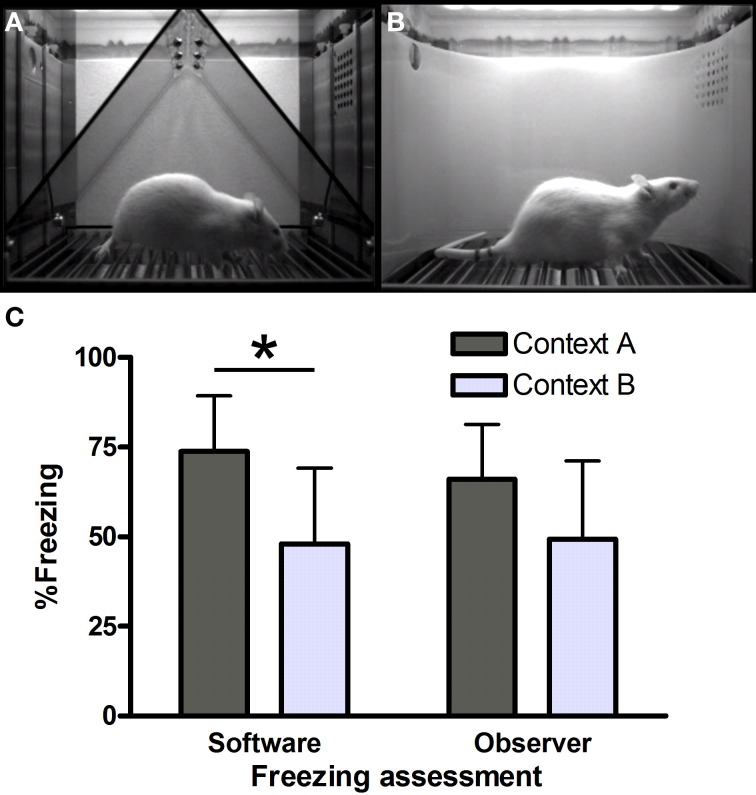
**Freezing in contexts (A) and (B)**. Screenshots from VideoFreeze software in **(A)** context A and **(B)** context B. Freezing **(C)** in contexts A and B in the third series of rats (*n* = 8 per group) as scored by software and human observers. ^*^Significantly different, unpaired *t*-test (*p* = 0.01).

On the first day, rats were trained in context A (Figure 1A). Four minutes after the start of the session, rats received five unsignaled footshocks (0.8 mA, 1 s), separated by 90 s. One minute after the last shock, animals were returned to their home cage. Twenty-four hours later, half of the rats were tested in context A and the other half in similar context B (Figure 1B). During this test, rats were exposed to the context for 8 min, without shocks. We hypothesized that there would be significantly less freezing (unpaired *t*-test) in context B than in A on day 2, because of a generalization decrement. The results presented here are part of an ongoing study, including other control groups, which will be discussed as a whole elsewhere. Freezing [absence of movement of the body and whiskers with the exception of respiratory motion (Fanselow, 1982)] was measured both manually [continuous measurement with a stopwatch from video recordings, cf. (Luyten et al., 2011, 2012)] and with VideoFreeze software (motion threshold 50, minimum freeze duration 30 frames) (Zelikowsky et al., 2012a). Percentage freezing was calculated as the percentage of time the rat was freezing during the 8-min test on day 2.

Context A (Figure 1A) consisted of a standard chamber (Med Associates), with a standard grid floor, a black triangular “A-frame” insert, illuminated by infrared and white light (intensity level 5) and cleaned and scented with a household cleaning product. Context B (Figure 1B) consisted of a standard chamber, with a staggered grid floor, a white plastic curved back wall insert, infrared light only and was cleaned and scented with another cleaner. Each chamber was located in one of two identical sound-attenuating boxes.

Rats were always trained in context A, and tested in either context A or context B. Previous (unpublished) data indicated that counterbalancing contexts A and B was not advisable because of different immediate post-shock freezing values (calculated by VideoFreeze) in both contexts when using the training protocol described above [52 rats trained in context A (average post-shock freezing 50%) vs. 24 rats trained in context B (31%), unpaired *t*-test *t*_(74)_ = 4.98, *p* < 0.0001]. The divergent freezing scores might be due to the different grids delivering the shocks. Given the findings in this paper, the difference may also be partially explained by a software scoring deviation.

## Results and discussion

Three consecutive series of rats (16 each, eight per group) were compared using software measurements and showed significant contextual discrimination between contexts A and B [A > B; series 1: *t*_(14)_ = 2.65, *p* = 0.02; series 2: *t*_(14)_ = 3.79, *p*< 0.01; series 3: *t*_(14)_ = 2.79, *p* = 0.01].

Series 3 (Figure 1C) was also scored manually, to allow comparison with yet another context (not described here) which was not located in a Med Associates box. Manual scoring was done by two independent observers, blind to the software scores. Surprisingly, hand-scored freezing (average of observers 1 and 2) did not yield significant differences between contexts A and B [*t*_(14)_ = 1.78, *p* = 0.10]. Moreover, software scores were significantly higher than manual scores in context A [74 vs. 66%, i.e., a 8% difference, paired *t*-test *t*_(7)_ = 3.93, *p* < 0.01], while there was virtually no difference between the software and manual scores in context B [48 vs. 49%, paired *t*-test *t*_(7)_ = −1.28, *p* = 0.24].

Because of this finding, we decided to examine inter-rater agreement and to retrospectively reevaluate freezing, also manually, in the two previous series of rats.

First, we investigated our findings in series 3 more thoroughly. The agreement between both human observers was substantial (Landis and Koch, 1977) [Cohen's kappa, a statistic to assess rater concordance, here using 20 ordered categories (0–5% freezing, 6–10%, etc.) = 0.65], with an average difference of 2.6% freezing. Therefore, we combined both ratings and used the average as the manual score. Correlations between software and hand-scored measurements were high in both contexts (93% in A and 99% in B), but while agreement in context B was substantial (kappa 0.71), it was only poor in context A (kappa 0.05). Additional analyses comparing software scores with those of a human observer for the two previous series of rats (16 rats per context) led to similar conclusions: only slight agreement in context A (kappa 0.18), but moderate agreement in context B (kappa 0.45).

To conclude, we find good agreement between software and manual scores in context B, but not in context A, while using identical software settings.

We therefore decided to reevaluate our camera calibration. Before the start of our studies, both cameras were calibrated in the base setup (without inserts) as described in the manual and discussed with experienced VideoFreeze users, and both cameras showed crisp and well-contrasted images (Figure 1). In addition, both cameras were calibrated using the “Calibrate-Lock” function before each rat. However, it is possible that differences in camera white balance (because of different plastic inserts used in both contexts) caused the observed discrepancies. To investigate this, we adapted the camera white balance in context A until it matched the other context (average grayscale intensity of 119, higher values led to very overexposed images), resulting in a slightly whiter image.

We trained and tested seven more naïve rats in context A and compared software scores with those of two human observers. Two additional rats were excluded from further analyses due to extremely low freezing (≤5%) on day 2. Unfortunately, adapting the white balance did not resolve the discrepancy between the software and observers' scores. Software scores were on average still 3% higher than human scores and when removing one outlying case (Grubb's test, *p* < 0.05) with an average observer score that was 23% higher than the software measure, this difference was even 8%. Analyses indicated that there was still poor agreement between software and observer scores (kappa 0.06).

In conclusion, changing the white balance did not improve agreement between software and manual scores in context A.

Given these findings, we decided to probe into other researchers' experiences and contacted seven research groups who recently (2011–2013) used the Med Associates software and setup for rat research and who implemented different context configurations in their papers. We asked which software settings they used and how they calibrated their systems. It turns out that there are considerable differences between various labs, as to how they define what the software should consider as freezing (Table 1).

**Table 1:** **Software settings and calibration procedures (adjustment of brightness, gain, and shutter) in seven research groups who use VideoFreeze software and several context configurations for rat conditioning studies**.

**Motion threshold**	**Minimum freeze duration (seconds)**	**Camera calibration**	**References**
18	1	In each context	Beeman et al., 2013
18	1	In standard context	Broadwater and Spear, 2013a,b
20	1.07	In standard context	Moffett et al., 2011
50^#^	1^#^	In standard context	Zelikowsky et al., 2012b
50 or 100	0.77	In each context	Long et al., 2011
120	1	In each context	Vander Weele et al., 2013
150^#^	3^#^	In each context	Sticht et al., 2012

Most information was obtained through personal communication with the authors. Settings indicated with # were mentioned in the corresponding publications.

All studies used several floors (different grids and/or plastic floors) and inserts (e.g., black A-frame and/or white curved back wall), as in our own experiments. The applied software settings were quite variable, with motion thresholds ranging from 18 to 150, and a minimum freeze duration of less than 1 s up to 3 s. Some authors used settings that were previously optimized for mice (Anagnostaras et al., 2010; Halladay et al., 2012; Beeman et al., 2013; Broadwater and Spear, 2013a,b), while others performed their own validations for rats (Zelikowsky et al., 2012b). Although not mentioned in their papers, several researchers reported to us that they optimized their parameters as well. J. Long used a 50 or 100 motion threshold depending on the context and in K. Goosens' lab, the motion threshold usually ranges from 100 to 150, depending on the context configuration and size and strain of the animal (but kept constant for all animals in a certain context on a given test day), and is determined by the experimenter (personal communication).

With regard to the calibration procedure, there was no uniformity either. While half of the research groups (including ours) calibrated the camera before the start of the experiments using a base context, the other half readjusted brightness, gain, and shutter in each context. A quick survey among two labs using VideoFreeze for mice gave a similar picture, with one of both calibrating in each context (Tayler et al., 2011) and the other at initial setup (McDermott et al., 2012). Although some authors mentioned to us that they found that camera calibration can greatly influence the measurements, we did not find meaningful improvements when adapting white balance in our experiments.

Taken together, there is considerable variability in the settings that are being applied by various research groups using the same software and equipment. It is somewhat surprising that rather divergent parameters are being put forward as optimal settings, although this might partially depend on the hand-scoring technique that was used for validation. A recent paper (Shoji et al., 2014) describing a new software package provides hints on how to determine motion thresholds and calibrate in each context. Nevertheless, the question remains whether studies and contexts become more comparable when using different, “optimized” settings in each context or if, conversely, this results in less commensurable measurements. Our finding that the agreement between manual and software measures varied substantially across contexts when using identical settings, is quite alarming. Thus, researchers should be aware of a potential divergence of agreement in different contexts when using a fixed motion threshold and minimum freeze duration. On the one hand, direct comparison of different contexts, e.g., in contextual generalization research, may lead to biased conclusions when using software measurements (in our case artificially increasing differences between contexts A and B). On the other hand, it is self-evident that manual scores by human observers also have drawbacks compared to the objectivity and time-efficiency of automated measurements. We feel that researchers should carefully compare both measurements and decide on the best choice for their research question.

Finally, we would like to stress that in the papers mentioned in Table 1, we see no interpretation problems, as most of these studies did not directly compare freezing between several contexts, and in case they did (Zelikowsky et al., 2012b), contexts were counterbalanced. Note however that, theoretically, even limited measurement deviations between contexts may induce heightened variability when counterbalancing contexts, thereby decreasing the chance of finding significant effects.

## Conclusion

While each researcher should balance the (dis)advantages of automated and manual scoring against one another, we believe that caution is required when using software measurements, particularly when comparing different context configurations or in case of subtle behavioral effects.

## Author contributions

Laura Luyten designed the experiments, partially conducted them, analyzed and interpreted all data and wrote the manuscript. Natalie Schroyens carried out part of the studies and contributed to the analyses and manuscript draft. Laura Luyten and Natalie Schroyens manually scored the freezing videos. Dirk Hermans and Tom Beckers contributed to the conceptual design and manuscript draft. All authors approved the final version of the manuscript.

### Conflict of interest statement

The authors declare that the research was conducted in the absence of any commercial or financial relationships that could be construed as a potential conflict of interest.
